# Characterization of Green Paints in Ming and Qianlong Dynasties’ Lin’xi Pavilion by Complimentary Techniques

**DOI:** 10.3390/molecules26020266

**Published:** 2021-01-07

**Authors:** Marcie B. Wiggins, Mengyu Liu, Catherine Matsen, Chang Liu, Karl S. Booksh

**Affiliations:** 1 Department of Chemistry & Biochemistry, University of Delaware, Newark, DE 19716, USA; mwiggins@udel.edu; 2 School of Architecture, Tsinghua University, Beijing 100084, China; desolatelin@gmail.com (M.L.); chang-liu@mail.tsinghua.edu.cn (C.L.); 3 Scientific Research & Analysis Laboratory, Winterthur Museum, Garden & Library, Winterthur, DE 19735, USA; cmatse@winterthur.org

**Keywords:** multivariate curve resolution, Raman, SEM-EDX, ToF-SIMS, polymorph, cultural heritage

## Abstract

During conservation of the painted ceiling decoration of Lin’xi Pavilion in the Forbidden City, two distinct paint campaigns were isolated as a unique case study into architectural paint materials during both the Ming and Qing dynasties. Paint samples and cross sections from both paint generations were analyzed with SEM-EDX, time of flight-secondary ion mass spectrometry (ToF-SIMS), XRD, FTIR, and Raman spectroscopies. Similar organic and inorganic materials characteristic of these time periods were identified. The pigments of interest found in both paint generations were botallackite and atacamite polymorphs. This suggests a shift from natural mineral sources to synthetic copper-based pigments for these larger architectural projects.

## 1. Introduction

**Figure 1:**
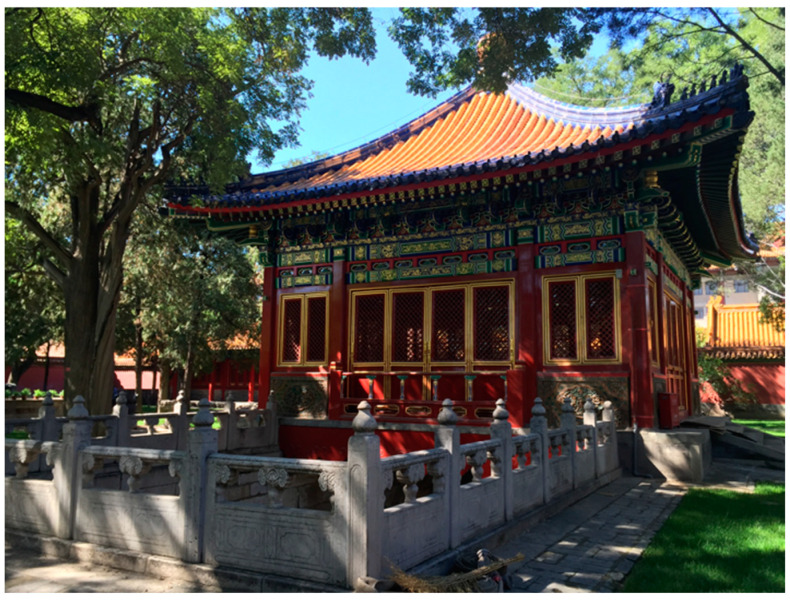
Exterior of Lin’xi Pavilion.

Lin’xi Pavilion (Lin Xi Ting) is a landscape building located in the southern part of the Cining Palace Garden, or the Garden of Compassion and Tranquility, found in the northwestern part of the Forbidden City, Beijing (Figure 1). It served as the residence and leisure area for the dowager empress and concubines of the Ming and Qing dynasties. The garden was built in the mid-1500s (Jiajing Era, Ming Dynasty), but Lin’xi Pavilion was constructed later. According to Sun Chengze’s *Chun Ming Meng Yu Lu*: “Lin Xi Hall was built in the 6th year of Wanli Era and was renamed as Lin Xi Ting in May of the 11th year of Wanli Era [1].” This corresponds to 1578, when the pavilion would have been completed. The *History of the Palace of the State* (*Guo Chao Gong Shi*), written in the 7th year of the Qianlong Era (1742), also referred to this building: “There is a pool in the garden and Lin’xi Pavilion is in front of the pool [2].” The building also appeared on a map of the Forbidden City in the Qianlong Era [3]. Thus, it can be inferred that the pavilion survived at its original location through the Ming and Qing Dynasties.

According to official historic archives of the Qing Dynasty, there were several restorations in the Cining Palace area during the Qing Dynasty. The largest renovation took place in the 30th year of the Qianlong Era (1765). In this year, Qianlong Emperor intended to restore Lin’xi Pavilion [4]. Additionally, there were some smaller maintenance projects during the 17–19th centuries [3].

As part of a recent conservation project, the decorated ceiling fabric of Lin’xi Pavilion was removed from the structure and moved to a conservation lab at the Department of Architectural Heritage at the Palace Museum. The existing ceiling decoration was separated into two paint layers, suggesting that it is comprised of two different paint campaigns (generations). Therefore, this object provided a good opportunity for historical study to compare the two generations of Ming and Qing painting. More details regarding sampling and analyses of the paints are provided by Wiggins [5].

**Figure 2:**
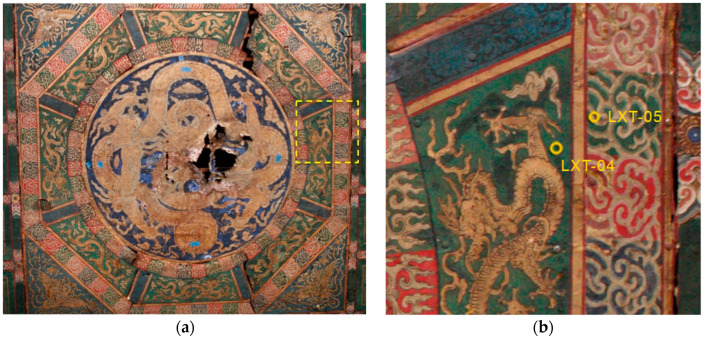
(**a**) The Lin’xi Pavilion ceiling painting (297 × 297 cm^2^) prior to treatment, where the yellow box indicates the region (**b**) where samples were located, indicated by yellow circles.

During treatment in 2015, the polychrome ceiling decoration of Lin’xi Pavilion was preserved according to extensive historical evidence. It can be deduced from the pattern style that the ceiling decoration was originally painted in the Ming Dynasty. However, the details of the paintings also have some distinctive features of mid-Qing Dynasty architectural paintings. The ceiling decoration contains various motifs, including dragons, phoenixes, clouds, and flames (Figure 2a). Green paints were predominantly found in the background of the dragon as well as in the green clouds and flames. Green paints from different areas are similar in color; they are not bright but rather have a saturated, pronounced, and uniformed appearance.

The identity of the green pigments used in this ceiling painting was of interest to conservators and art historians. The literature on artists’ materials from the 16th–18th centuries suggest that green pigment sources were either “mineral green” or “copper green”. The terms “mineral green” and “mineral blue” were used for mined basic copper carbonates, malachite (Cu_2_CO_3_(OH)_2_) and azurite (Cu_3_(CO_3_)_2_(OH)_2_), while “copper green” was an umbrella term for other copper-based pigments, such as verdigris (Cu(CH_3_COO)_2_ H_2_O), atacamite (Cu_2_Cl(OH)_3_), etc. [6,7]. Determining which pigment was used in this ceiling painting informs on the artists’ materials that would have been available and used for this large-scale architectural project in both the Ming and Qing dynasties. The identity of pigments used can inform conservators and historians of the source and cost of the ceiling paint materials.

In order to identify and study these materials, the authors first utilized scanning electron microscopy-energy dispersive x-ray spectroscopy (SEM-EDX) and time of flight-secondary ion mass spectrometry (ToF-SIMS) to map the elemental and molecular fragment distributions across the cross sections. These techniques have been established in the field to identify layered materials within paintings and other cultural heritage objects, first for inorganics and then for organics [8,9,10]. Following these analyses, characterization of pigment particles and some organic material was carried out using X-ray diffraction (XRD), and infrared and Raman spectroscopies, with specific interest in the green pigments. Again, these methods are commonplace in studying cultural heritage objects for the identification of artists’ materials to guide further treatments and studies.

## 2. Results

**Figure 3:**
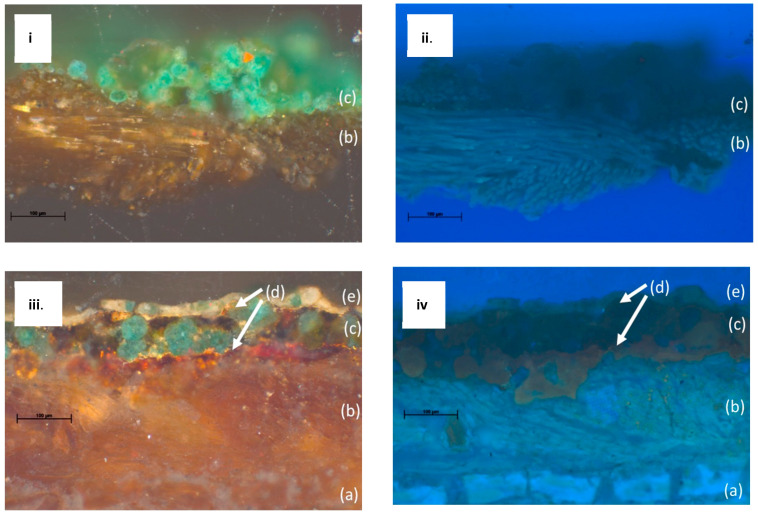
Cross-sectional images of LXT-04 (**i**) and LXT-05 (**iii**) in normal light and LXT-04 (**ii**) and LXT-05 (**iv**) in ultraviolet light: the layered structure of both the first and second generations is (a) the paper support, (b) the silk substrate, (c) green paint, (d) the gold leaf, and (e) white paint.

To establish the layered structure of the paintings, the cross sections were examined and documented (Figure 3). In both LXT-04 and LXT-05, a textile substrate is clearly seen just below the paint layer, shown in Figure 3b. With LXT-05, the cellulosic paper support, shown in Figure 3a, is seen below the textile substrate. This sequence corroborates the abovementioned process of the second-generation paint and textile being applied to a paper support prior to being adhered to the ceiling. The green paint layers in both generations contained spherical green particles, approximately 40 µm in diameter. Specific to the newer generation (LXT-05), thin layers of gold leaves (Figure 3d) exist both above and below the green paint layer. Also, the upper most paint layer is thin (approximately 20 µm) and white (Figure 3e). A few small green particles are noted in the white layer; the origin could be intentional or an artifact of micro-toming the paint. With ultraviolet light illumination, an organic material, such as shellac or a natural resin, just below the green paint layer is made evident by its orange fluorescence in some areas of the cross section. It is more pronounced in regions with fewer green pigment particles above it (not shown).

**Figure 4:**
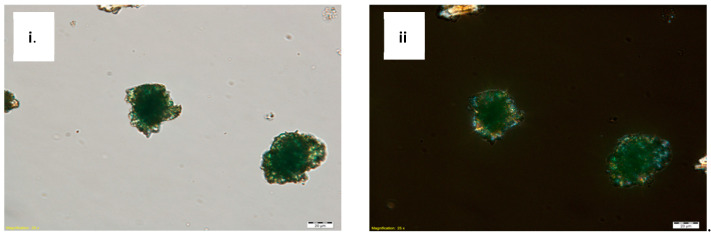
Dispersed sample of the pigments from LXT-04 at 500× magnification in (**i**) plane polarized transmitted light and (**ii**) crossed-polarized light.

Scrapings from the pigmented layers were analyzed with polarized light. In plane-polarized light, the pigment particles appear as a fine mesh of translucent green, rounded crystals. They were in high relief with a lower Refractive Index (RI) than the medium (Figure 4i). Under crossed polars, the samples have moderate birefringence. Some particles appear to be second-order interference colors (Figure 4ii), which indicates the presence of atacamite [11,12].

**Figure 5:**
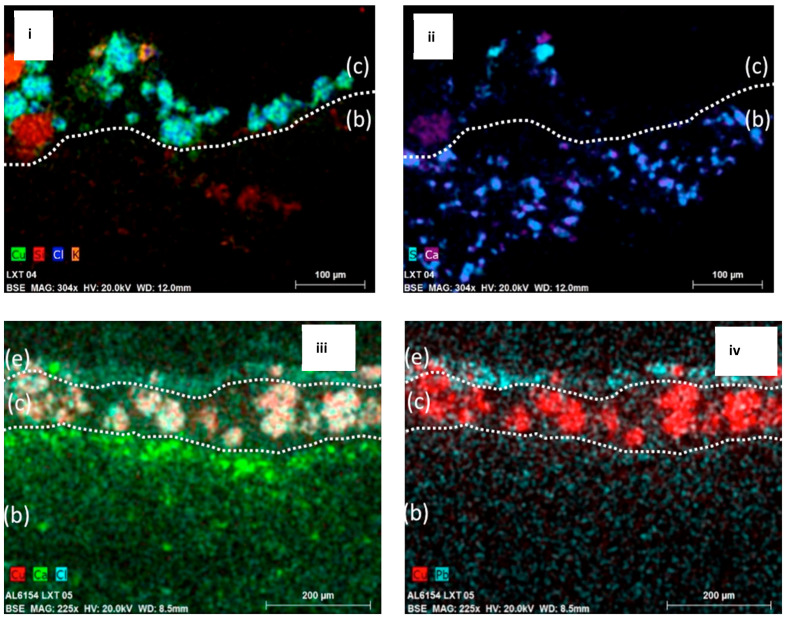
SEM-EDX false color elemental maps of elements for LXT-04 (**i**,**ii**) and LXT-05 (**iii**,**iv**) with layers for silk (b), green paint (c), and white paint (e).

The cross sections were studied with SEM-EDX to determine the elemental distribution across the layers (Figure 5). The data show the presence of copper and chlorine mapping together within the pigment particles from the two generations. In both samples, calcium and sulfur were detected within the textile fibers. In addition, for LXT-04, potassium and silicon were mapped within the areas of some slightly transparent particles in the paint layer. For LXT-05, the upper white paint layer is rich in lead.

**Figure 6:**
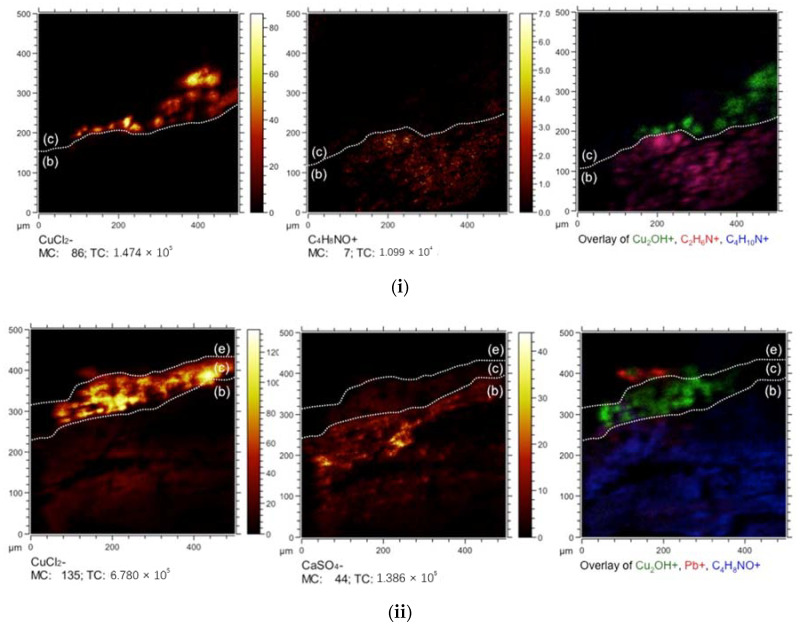
Time of flight-secondary ion mass spectrometry (ToF-SIMS) maps of mass fragments for LXT-04 (**i**) and LXT-05 (**ii**).

To identify molecular species in the stratigraphies, ToF-SIMS was then used to map mass fragments in the cross sections. The mass fragment CaSO_4_^−^, found in the fiber substrates in negative mode, indicates a calcium sulfate ground and is consistent with the EDX elemental maps of both paint generations (Figure 6). The presence of copper chloride trihydroxides was identified in the green paint layer by the presence of CuCl_2_^−^, CuCl^−^, Cl^−^, Cu*_n_*^+^, and Cu_2_OH^+^ fragments. These fragments correspond to the pigment particles in both cross sections, which have previously been attributed to the colorant atacamite by Richardin et al. [13] and by Kim et al. [14]. Atacamite is a common copper chloride trihydroxide pigment, but ToF-SIMS cannot determine the specific polymorphs alone.

Additionally, positive amino acid mass fragments, glycine (CH_4_N^+^), alanine (C_2_H_6_N^+^), proline (C_4_H_8_N^+^), and valine (C_4_H_10_N^+^ and C_5_H_7_O^+^), were detected in the substrate region due to the silk textile. The silk fibers, composed of fibroin, contain glycine, alanine, and serine [15]. The presence of the remaining amino acids and the unique hydroxyproline mass fragment (C_4_H_8_NO^+^) indicates that the source of amino acids in the lower layers is also from an additional protein: animal glue [9]. The source of hydroxyproline was not the silk substrate but a fish or mammalian glue [16]. This practice agrees with known artistic methods for ceiling fabric paintings [17]. Trace amounts of fatty acid markers, such as for palmitic acid (C_16_H_31_O_2_^−^), were found, but this is likely from the glue. However, organic fragment markers for protein and oils were not detected in regions that correspond to the green and white paint layers in the cross sections [8,9]. Therefore, the paint binder could not be directly identified.

**Figure 7:**
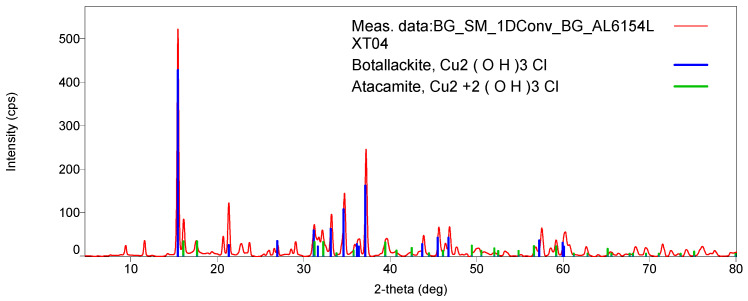
XRD patterns of LXT-04 and the references botallackite and atacamite.

**Figure 8:**
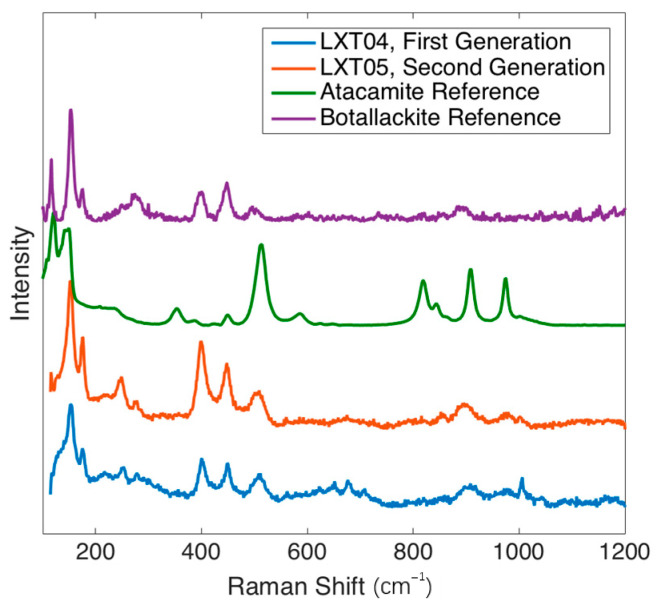
Raman spectra of LXT-04 and LXT-05 compared to reference spectra of atacamite and botallackite.

XRD and Raman spectroscopy were used to specify the copper chloride trihydroxide polymorph (Cu_2_(OH)_3_Cl). XRD patterns for both LXT-04 and LXT-05 show a mixture of botallackite and atacamite crystals [18]. Both are polymorphs, although botallackite is less common in nature [7,19,20]. Figure 7 shows the botallackite and atacamite comparison of XRD patterns for LXT-04. The presence of botallackite was verified as well with Raman spectroscopy [21]. Compared to the RRUFF database (http://rruff.info) standard for botallackite (R070066), characteristic bands at 400, 450, and 510 cm^−1^ were found in both samples. Additionally, weak characteristic bands at 820, 910, and 975 cm^−1^ for atacamite (R050098) were also found in LXT-04 (Figure 8).

## 3. Discussion

### 3.1. Organic Materials

The organic materials were able to be identified spatially on the cross sections using ToF-SIMS and with supplemental verification on isolated materials by GC-MS and FTIR. Both generations of paint were applied to the ceiling on a textile substrate, which has been identified as silk. Both of these silks were treated with a common ground material, calcium sulfate, and animal glue as a binder. The cross section of the second generation, LXT-05, shows the paper substrate that was placed in between the two generations of paint [17].

However, neither paint layer shows any unique mass fragments for protein or oil. With GC-MS (not shown) and ToF-SIMS, fatty acids were identified, but these are likely trace amounts resulting from animal glue.

The microscopic analysis using ultraviolet light showed one material in the second generation of paint having orange fluorescence. This phenomenon suggests the presence of shellac just below the paint layer. FTIR verified a natural resin on the back of the fibers based on a carbonyl band at 1708 cm^−1^ and C–H stretching at 2930 and 2857 cm^−1^ [22]. However, the presence of shellac was not verified with GC-MS (not shown). Shellac is surprising to find as a material used in second-generation, 17th century paint [23,24]. It is possible that this natural resin found at the edge of the cross section with little to no paint was part of a later restoration. A more definitive identification of this resin requires further study.

### 3.2. Inorganic Materials

Calcium sulfate was detected within the silk substrate and served as a ground material for paint layers. This practice is consistent with traditional painting on silk from the 15th to 18th centuries [17].

The ceiling was predominantly painted green in both the first and second generations. Based on the previously mentioned analyses with Raman spectroscopy and XRD, the Lin’xi Ting ceiling is painted as a mixture of botallackite and atacamite in both generations. To these authors’ best knowledge, botallackite has rarely been identified in Chinese architectural painting. Botallackite has been found in wall paintings in Furi Temple in Gansu Province and in the Five Northern Provinces’ Assembly Hall in Shaanxi Province [11,25]. Atacamite and another polymorph, paraatacamite, have also been found in Chinese works [6,26]. Previously, botallackite was found along with atacamite in cave murals [27,28]. The challenges in identifying botallackite previously were (1) the low sample population of Chinese architecture, (2) the difficulty to differentiate botallackite and atacamite visually or with PLM, (3) the lack of botallackite reference Raman spectra, and (4) the small sample size for XRD [11]. As a result, copper chloride crystals cannot always be correctly distinguished as atacamite or botallackite and, more commonly, the copper chloride trihydroxide crystals are categorized as only atacamite or “copper green”. Because of this, there is likely more botallackite present in Chinese architectural paints than is currently documented.

The identification of both botallackite and atacamite in the 16th (first generation) and 18th (second generation) centuries in Lin’xi Pavilion indicates the historical usage of “copper green” pigments. The term “copper green” refers to many types of copper-based pigments, such as copper chlorides and copper acetates (commonly referred to as verdigris) [6]. Alternatively, historic green and blue pigments are referred to as “mineral green” (Shi lu) or “mineral blue” (Shi qing) for basic copper carbonates, malachite (Cu_2_(CO_3_)_2_(OH)_2_) and azurite (Cu_2_CO_3_(OH)_2_), respectively. Mineral green was commonly used before the 18th century in Chinese architectural painting [6]. “Copper green” pigments also appeared at that time, but after the 18th century, they were more common than “mineral green” [12]. In this case study, both generations from the 16th and 18th centuries lack any “mineral green” pigments, and there is only atacamite and botallackite used for the large ceiling paintings. The mineral reserves of malachite decreased with time, which caused the price to increase. Hence, craftsmen would have been more inclined to use copper green instead. Primary literature suggests that the prices for “copper green” were cheaper than for “mineral green” [29], and since a large quantity of the pigment would be needed, the less expensive option was chosen. During the 15th century, recipes for synthesizing copper chloride trihydroxide pigments appear in the literature, which is the likely cause for decreased costs and increased usage [6]. Currently, our group is carrying out further studies into the copper chloride trihydroxide compositions during this transition period of the Ming and Qing dynasties [30].

The two generations of green paint are similar in composition, but the first generation of paint (LXT-04) contains silicon particles equal in size to the copper-based particles. The SEM-EDX image also shows smaller potassium particles above the green paint particles. This is possibly a result of dirt accumulated over the years and then trapped under the second generation of paper, silk, and paint. It is also possible that silicon and/or potassium were originally additives to the green paint layer.

The second generation of paint (LXT-05) involved more decoration, which can be seen in the layers of white paint and gold leaf. From the microscopic images, the thin layers of gold were identified both above and below the green pigment particles. This corresponds to the golden cloud motif on the edges of the ceiling design. The thin, lead, white layer was mixed with some green pigment to lighten it and was painted directly on top of the upper most gold. This lighter outline around the motif is referred to as Tuiyun. This resulted in a more decorative and elaborate second-generation ceiling of Lin’xi Pavilion.

## 4. Materials and Methods

The two generations have a similar structure: a supporting substrate layer, followed by a paint layer, and finally a gilded layer in certain areas, as seen in Figure 3. The supporting substrate layer was made of many layers of paper and textile pasted together. After painting and gilding, the entire sheet was pasted on a wooden panel and then installed onto the timber structure of the building. This is a traditional technique to make ceiling decorations in Chinese architecture [17]. In the Qing dynasty restoration campaign (c. 1765), the second-generation supporting layer was made of silk and paper and then painted. The goal was not to remove old layers but to paste the new supporting layer onto the old surface, thus creating the second-generation painting on top of the first generation, which is how it remained until the 21st century [31]. As part of that addition, the same patterns were painted on the new supporting layer with some slight variations. In some later, small-scale conservation efforts, patches were made on the second generation of painting to cover damage and deterioration [31].

Two green paint samples were taken from the east side of the ceiling from adjacent areas but from different generations for comparison. The samples were cast separately in mini-cubes, of approximately half inch widths, with polyester resin (Extec polyester clear resin (methyl methacrylate monomer) with a methyl ethyl ketone peroxide catalyst (10 mL: 8 drops), Extec Corporation^®^, Enfield, CT, USA). The resin was allowed to cure for 24 h at room temperature and under ambient light. Excess casting medium was removed from the cube just up to the surface of the sample with a jeweler’s saw (Rio Grande saw blades, laser gold). The cubes were then dry, hand-polished successively with 400- and 600-grit Buehler Carbimet paper (silicon carbide) and 1500- to 12,000-grit Micro-Mesh Inc. polishing cloths (silicon carbide or aluminum oxide) to expose the cross section. Sample LXT-04 was taken from the first-generation paint layer of the ceiling at an area of loss of the second-generation that exposed the underlying, original painting (Figure 2b). Sample LXT-05 was taken from an area of green and gilded clouds (Figure 2b) from the second-generation painting surface, corresponding to the later 18th c. restoration layer.

### 4.1. Visible and Ultraviolet Microscopy

To establish finished stratigraphies of the paint cross sections, from the abovementioned sample locations, samples were examined and digitally photographed using a Zeiss Axio Imager M2m binocular microscope (20× objective) equipped with a Kübler Codix HXP 120C mercury lamp for reflected visible and ultraviolet light (Carl Zeiss Industrielle, Oberkochen, Germany). The samples were viewed in a dark field with reflected light and by using the Zeiss 02 cube (excitation 365 nm, barrier 420 nm, beam splitter 395 nm). Images were taken with the Zeiss AxioCam HRc digital camera using Zeiss AxioVision software (v.4.9.1.0) (White Plains, NY, USA).

### 4.2. Polarized Light Microscopy (PLM)

Polarized light microscopy (PLM) is a common method of pigment identification based on the optical properties of the crystals. For PLM, pigment particles were scraped from the bulk paint with a scalpel, transferred to a glass slide, and covered with a coverslip. The heat-reversing mounting medium, Meltmount™ (refractive index of 1.662 at 25 °C) (Cargille Laboratories, Cedar Grove, NJ, USA), was melted at approximately 70 °C and then injected between the cover slip and the slide. After cooling, the pigment particles dispersed in the mounting media. A Nikon LV100ND microscope with a polarized attachment and a DS-Ri2 microscope camera were used for sample observation and photomicrography, using NIS-Elements software (Nikon Instruments, Mellville, NY, USA). 

### 4.3. SEM-EDX

For SEM-EDX analysis, the cross sections were mounted to an SPI Supplies Zeiss aluminum slot head stub (12.7 × 3.1 mm^2^) with SPI Supplies double-sided carbon tabs (12 mm diameter). (SPI Supplies, West Chester, PA, USA) SPI Supplies conductive carbon paint (20% *w/w* colloidal graphite in isopropanol) was applied on the side and top surfaces of the casting media, without covering the cross-sectional surface itself to prevent charging effects. The samples were examined using a Zeiss EVO MA15 scanning electron microscope with LaB_6_ source at an accelerating voltage of 20 kV for the electron beam, a working distance of approximately 8 mm, and a sample tilt of 0° (Carl Zeiss Industrielle, Oberkochen, Germany). The EDS data were collected with the Bruker Nano X-flash^®^ detector 6|30 and analyzed with Quantax 200/Esprit 1.9 software (Bruker Corporation, Billerica, MA, USA). 

### 4.4. Time of Flight-Secondary Ion Mass Spectrometry

As previously established by Voras, et al. [9]. and deGhetaldi, et al. [8], a ToF-SIMS IV with the upgraded capabilities of a ToF-SIMS V (ION-TOF USA Inc, Chestnut Ridge, NY, USA) was used to image the paint cross sections. A bismuth/manganese primary ion source was used to collect all images and spectra in the analysis chamber with a pressure of 5.0 × 10^−8^ mbar or less. The mode consisting of 25 keV Bi_3_^+^ clusters of a pre-bunched pulse width of 640 ps and target current of approximately 0.18 pA was used. The image pixel density was 128 × 128 pixels with the ion dose density set to the static SIMS limit of 1 × 10^12^ ions/cm^2^. At the static SIMS limit, <0.1% of the sample surface was removed or damaged [32]. To dissipate charge buildup on the sample surface, a low-energy (75 eV) electron flood gun was used. Each mass spectrum was calibrated using ubiquitous ions for both positive and negative ion modes. The positive ion mass calibrations used were H^+^, H_2_^+^, H_3_^+^, C^+^, CH^+^, CH_2_^+^, CH_3_^+^, C_2_H_3_^+^, C_3_H_5_^+^, C_4_H_7_^+^, C_5_H_5_^+^, C_6_H_5_^+^, and C_7_H_7_^+^. The negative ion mass calibrations used were H^−^, H_2_^−^, C^−^, CH^−^, CH_2_^−^, CH_3_^−^, C_2_^−^, C_2_H^−^, C_3_^−^, C_4_^−^, C_5_^−^, C_6_^−^, and C_7_^−^. ION-TOF Measurement Explorer (version 6.2) software was used for all collection and data analysis, and the ion images were normalized by total ion intensity.

### 4.5. X-ray Diffraction (XRD)

XRD was carried out using a Rigaku D/max Rapid II diffractometer with a copper anode X-ray tube and 0.3-mm collimator (Rigaku Corporation, Tokyo, Japan). Isolated material from the original paint layer (LXT04) was adhered to a glass loop by Parabar 10,312 liquid (Hampton Research, Aliso Viejo, CA, USA) and secured to the sample stage. The sample was analyzed by rotating phi (0–360° rotation) at a speed of 10°/s with omega held at 0°. Collection time was 3 h with an X-ray tube held at 40 kV and 30 mA. The XRD pattern for the second-generation layer (LXT05) was collected from the cast cross section. The X-ray beam (45 kV and 40 mA) was focused on the green paint layer of the cross section, and the sample was oscillated phi from 42 to 49° at a speed of 1°/s with omega held at 45° for a collection time of 8 h. Rigaku RAPID/XRD software (v.2.4.2) (The Woodlands, TX, USA) was used for instrument operation and data collection, and Rigaku 2DP software (v.2.0.1.1) was used to select the portion of diffraction rings for interpretation. Rigaku PDXL 2 software (v.2.3.1.0) was used to interpret the diffraction pattern, and the Powder Diffraction File from the International Center for Diffraction Data (ICDD) was used as a reference database.

### 4.6. Raman Spectroscopy

Raman measurements were performed using a single grating Invia Renishaw spectrometer coupled with a diode laser source emitting light at 785 nm, determining a spectral resolution of about 3 cm^−1^ (Renishaw Inc., West Dundee, IL, USA). The cross-sectional samples were analyzed with a 50× magnification objective, which provides a laser spot with about 2 μm diameter. The spectra were recorded in the range 100–1400 cm^−1^, with the laser power on the sample at 1% of the total laser power, 500 mW, and an acquisition of 30 s, with 3 accumulations. Spectra were collected using Renishaw WiRE 3.4 software (Renishaw Inc., West Dundee, IL, USA).

### 4.7. Fourier-Transform Infrared Spectroscopy (FTIR)

The Fourier-transform infrared microspectroscopy (FTIR) sample was acquired with a stainless-steel scalpel from the backing silk of a paint scraping from LXT05 with the aid of a stereomicroscope and then placed directly on a diamond cell. The material was rolled flat on the cell with a steel micro-roller to decrease thickness and to increase transparency. The sample was analyzed using the Thermo Scientific Nicolet 6700 FT-IR with Nicolet Continuμm FTIR microscope in transmission mode (Thermo Fischer Scientific, Waltham, MA, USA). Data were acquired for 128 scans from 4000 to 650 cm^−1^ at a spectral resolution of 4 cm^−1^. Multiple scrapings of the sample were taken from the sample, and multiple spectra were taken from different areas within each scraping. Spectra were collected with Omnic 8.0 software and analyzed in this program with various Infrared and Raman Users Group (IRUG; (http://irug.org/)and commercial reference spectral libraries.

## 5. Conclusions

Using SEM-EDX, ToF-SIMS, FTIR, Raman, and XRD, an extensive characterization of the Lin’xi Ting ceiling painting documented the materials used in both the Ming and Qing dynasty campaigns. The materials were very similar across the two paint generations. Both contained silk and paper substrates which had been treated with calcium sulfate and animal glue. The green paint pigments were identified as a mixture of atacamite and botallackite polymorphs. The second paint generation included gold leaves and lead white paint.

The presence of atacamite and, predominantly, botallackite opens a discussion regarding copper-based green pigments used in China at this time. As atacamite and botallackite would be considered “copper green”, this suggests that this was a more common material for architectural painting during the Ming and Qing dynasties than was previously thought. Malachite would have decreased in usage during this time period, and new syntheses of atacamite and botallackite suggest that they would have been a cheaper, preferred option for craftsmen. Currently, additional investigations are underway to explore the increased synthesis and usage of atacamite and botallackite in Chinese architectural paints.

## Data Availability

The data presented in this study are available on request from the corresponding author.

## Sample Availability

Archived spectra of the historical paint samples are available from the authors.
